# A Fast Algorithm for Intra-Frame Versatile Video Coding Based on Edge Features

**DOI:** 10.3390/s23136244

**Published:** 2023-07-07

**Authors:** Shuai Zhao, Xiwu Shang, Guozhong Wang, Haiwu Zhao

**Affiliations:** School of Electrical Engineering, Shanghai University of Engineering Science, Shanghai 201620, China; m025120510@sues.edu.cn (S.Z.);

**Keywords:** versatile video coding (VVC), fast algorithm, CU partition, edge direction

## Abstract

Versatile Video Coding (VVC) introduces many new coding technologies, such as quadtree with nested multi-type tree (QTMT), which greatly improves the efficiency of VVC coding. However, its computational complexity is higher, which affects the application of VVC in real-time scenarios. Aiming to solve the problem of the high complexity of VVC intra coding, we propose a low-complexity partition algorithm based on edge features. Firstly, the Laplacian of Gaussian (LOG) operator was used to extract the edges in the coding frame, and the edges were divided into vertical and horizontal edges. Then, the coding unit (CU) was equally divided into four sub-blocks in the horizontal and vertical directions to calculate the feature values of the horizontal and vertical edges, respectively. Based on the feature values, we skipped unnecessary partition patterns in advance. Finally, for the CUs without edges, we decided to terminate the partition process according to the depth information of neighboring CUs. The experimental results show that compared with VTM-13.0, the proposed algorithm can save 54.08% of the encoding time on average, and the BDBR (Bjøntegaard delta bit rate) only increases by 1.61%.

## 1. Introduction

In recent years, with the development of information technology, multimedia technologies such as 4K ultra-high-definition videos, 360-degree immersive multimedia, and high-dynamic-range videos have rapidly developed, resulting in a rapid and sharp increase in data volume. This puts tremendous pressure on data storage and transmission, and the previous high-efficiency video coding standard, high-efficiency video coding (HEVC), has difficulty meeting the compression requirements [1].

**Figure 1:**
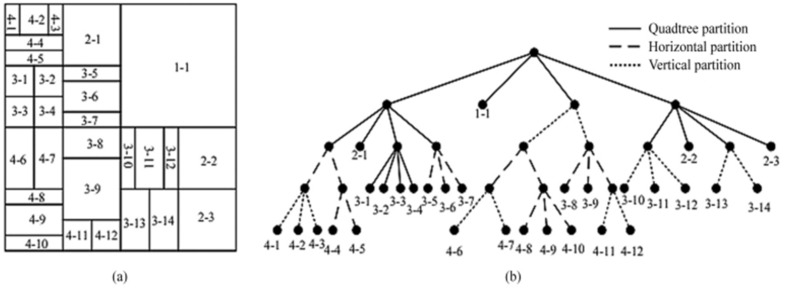
Example of the CU partition structure in VVC. (**a**) example of the partition structure obtained after recursive traversal; (**b**) the corresponding tree structure.

To overcome this difficulty, the Joint Video Coding Expert Group began exploring the next-generation video standard, and developed a new video coding standard called Versatile Video Coding (VVC) [2]. The goal of VVC is to achieve compression efficiency improvement of over 50% while maintaining the same video quality as the HEVC. VVC introduces a new segmentation technique called Quadtree with Nested Multi-Type Tree (QTMT) [3]. In addition to the QT partition structure, there are four other multi-type tree (MTT) partition structures, including vertical binary tree (VBT), horizontal binary tree (HBT), vertical ternary tree (VTT), and horizontal ternary tree (HTT). Introducing QTMT makes the shape of coding units (CU) more flexible and diverse, which can increase the number of CUs that need to be recursively traversed during the rate-distortion optimization (RDO) process. Figure 1a shows an example of the partition structure obtained after recursive traversal, and Figure 1b shows the corresponding tree structure. Under the condition of all intra configuration, the average complexity of VVC is 18 times that of HEVC [4], which affects the application of VVC in real-time encoding scenarios.

To solve the problem of high computational complexity in the partition process of VVC, a fast partition method based on edge features is proposed. The main contributions of the proposed method are as follows:

(1) Using edge information as the basis for selecting partition modes leads to more accurate results compared to using texture complexity as the basis for selecting partition modes.
(2) We propose a method for calculating the feature value of the edge, which is exploited to predict the partition pattern.
(3) The partition information and texture complexity of adjacent CUs are utilized to determine whether to terminate the current CU partition, resulting in more accurate results.

## 2. Related Work

Currently, research on fast algorithms for VVC can be divided into two parts: machine learning-based and coding content-based methods.

For machine learning-based methods, Fu et al. [5] propose a fast decision binary partition algorithm based on Bayesian rules by utilizing the correlation between the current CU and the sub-CU after horizontal binary segmentation to terminate vertical binary segmentation. Li et al. [6] propose a multi-stage exit CNN (MSE-CNN) model with an early exit mechanism to determine the CU partition. Then, an adaptive loss function is designed to train the MSE-CNN model. Zhang et al. [7] divide a CTU into four parts to train a neural network and calculate the probability of various partition modes in a coding tree unit (CTU). Once the probability of the partition mode exceeds the threshold, it will be skipped to achieve complexity reduction. In [8], Zhang et al. design a fast coding unit (CU) partition and an intra-mode decision algorithm, which uses a random forest classifier (RFC) model to achieve a fast partition of the CU. Yang et al. [9] transform the QTMT partition process into multiple binary classification problems for each decision layer, which is processed by a decision tree classifier. Although this method saves coding time, BDBR (Bjøntegaard Delta Bit Rate) losses are also high. In [10], Wu et al. propose a Hierarchical Grid Fully Convolutional Network (HG-FCN) framework to predict level-specific partition structures, achieving a certain degree of complexity reduction. Saldanha et al. [11] use a classifier to make early decisions in Direct Component (DC) mode and Planar mode during the prediction process, and use the pixel variance of sub-blocks to decide whether to use intra-frame sub-block partition technology. In [12], a convolutional neural network is trained to predict partition modes by training a probability vector. In [13], a neural network model is trained using cross-entropy and is used for early termination of the partition process. In [14], a fast algorithm based on machine learning is proposed, which uses texture complexity to determine the division direction and a lightweight neural network to determine the division mode. In [15], the authors present a low-complexity method, which is formed of five binary Light Gradient Boosting Machine (LightGBM) classifiers. In [16], a fast CU partitioning decision algorithm is presented based on texture complexity and convolutional neural networks (CNNs). It utilizes a symmetric convolution kernel to extract features and redesigns the loss function.

For coding content-based methods, Zhang et al. [17] propose a fast partition scheme based on adjacent sub-regions, which skips unnecessary partition modes in advance based on the similarity of adjacent sub-regions in the horizontal and vertical directions. In [18], Song et al. measure the texture complexity in the vertical and horizontal directions according to the sum of the mean absolute deviation (SMAD) of the sub-blocks. On this basis, unnecessary division modes are skipped. In [19], Li et al. use the gradient of the Scharr operator to describe texture information, and use the edge differences of sub blocks to describe structural information. On this basis, they propose a fast algorithm based on texture features. In [20], Shang et al. make a rapid decision on the CU partition process by utilizing the partition mode and size distribution of adjacent CUs, and optimize the decision-making process of inter-frame prediction modes. In [21], Zhang et al. use corner features and average color brightness differences to classify screen content. Then, for different screen contents, they exploit different strategies to predict the coding mode. In [22], Fan et al. use a Sobel operator to calculate the gradient of a CU and terminate the QT partition based on the gradient. Then, texture information is used to measure the differences between partition structures, based on which partition patterns are determined. Shang et al. [23] predict the quadtree division depth of the current CU in advance by analyzing the correlation between adjacent CUs. Additionally, image texture features are utilized to make early decisions about the MTT division process. In [24], Zhang et al. determine whether to split using CU texture information, and skip unnecessary partition modes according to the distribution of the residual coefficients. In [25], a latitude-based preprocessing is introduced to enable early termination of the coding unit (CU) partition in the polar region. In [15], the authors apply the factor of average background luminance for just-noticeable distortion to identify the visually distinguishable (VD) pixels within a coding unit (CU).

Although the above methods achieve some complexity reduction, they do not achieve a good balance between complexity reduction and compression performance loss. Complexity reduction that maintains good compression performance is limited, and the improvement in the application of VVC in real-time scenarios is not significant. Compression performance with higher complexity reduction has a greater loss, and cannot meet the coding requirements.

## 3. Proposed Method

### 3.1. Principle

In the final partition results of VVC, the MTT partition structure accounts for almost one-third of all partition structures. It is the most complex operation, averaging over 90% of the total encoding time [10]. In the MTT partition decision process, the choice of partition mode has great flexibility, but only one of the vertical or horizontal directions is used in the final decision. Therefore, if we can skip unnecessary MTT partition modes in advance, we can effectively reduce the time complexity of intra-frame coding.

From the partition results, some CUs tend to have larger partition sizes. However, the encoding process still exhaustively searches all CU size options to find the optimal mode. If the partition process for these CUs can be terminated in advance, it will also effectively reduce the time complexity.

**Figure 2:**
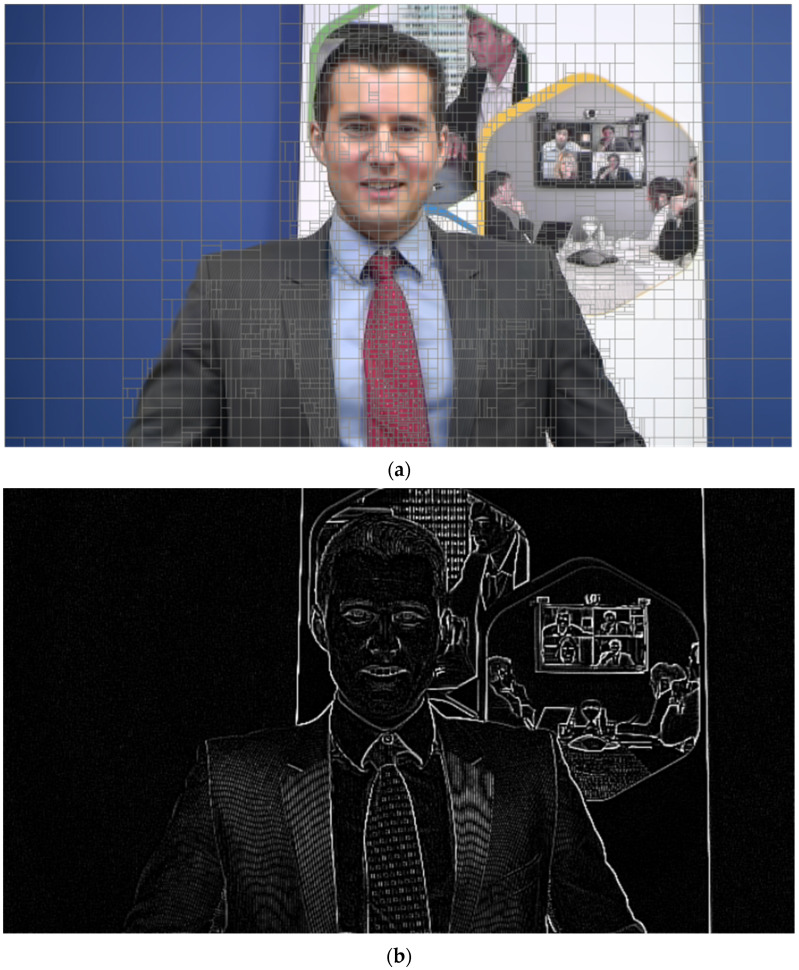

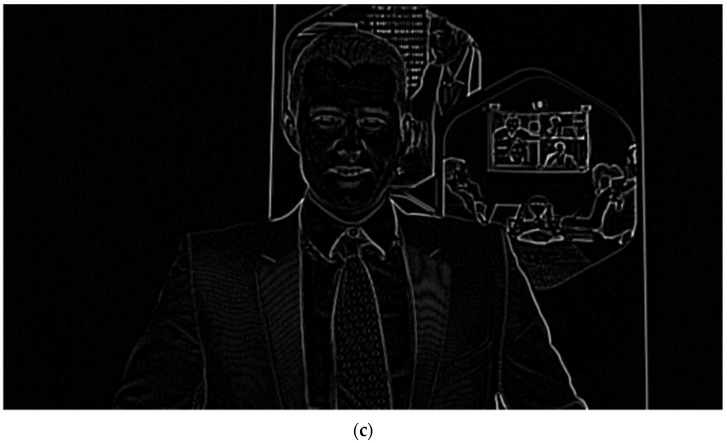
(**a**) The partition result for Johnny under QP = 32. (**b**) The edge extraction for Johnny with σ = 0.1. (**c**) The edge extraction for Johnny with σ = 2.1.

Figure 2a shows a CU partition result for the sequence Johnny in VVC intra coding. By observing Figure 2a, it can be seen that for regions containing edges, smaller partition sizes are usually adopted to adapt to complex texture directions and achieve better compression efficiency. In addition, the selection of partition mode is related to the direction of the edge. In contrast, regions without edges have simpler textures, and larger partition sizes are usually adopted. Inspired by this, we divided the encoding frames into two categories: complex-texture areas with edges and simple-texture areas without edges. For complex-texture areas, edge features are computed to skip unnecessary partition modes, while for-simple-texture areas, the partition process is terminated in advance.

### 3.2. Edge and Edge Feature Extraction

Traditional edge detection operators such as the Sobel operator [26] are based on first-order derivatives to determine the edges of an image. However, first-order derivatives only reflect the velocity of pixel changes in an image, and cannot prove the presence of edges, leading to inaccurate edge detection and localization. The Canny operator [27] has higher accuracy in edge detection than the Sobel operator, but it still has errors and high computational complexity, which goes against our intention to reduce the computational complexity. The Laplacian of Gaussian (LOG) operator [28] is based on second-order derivatives to determine the edges of an image. Second-order derivatives reflect the degree of frequency changes (discontinuity) in the pixel change curve of an image, and can detect more details. Additionally, the extraction of edge positions is more accurate, overcoming the shortcomings of previous operators. Additionally, the LOG operator uses a Gaussian filter to smooth the image before edge detection [29], which eliminates noise interference. Therefore, we chose the LOG operator to extract the edges of the image and used the results to calculate edge features.

The LOG operator has accurate edge position detection and can extract more complete edges. Before extracting the edges, the Gaussian filter is used to remove the noise in the image. The setting of the standard deviation threshold of the Gaussian distribution during the filtering process affects the edge extraction effect. As shown in Figure 2b, if the value is set too low or is not set, there will be a lot of noise in the extracted edges. If the value is set too high, some edge information may be lost. To obtain the best value, we designed the following experiment.

The first step was to calculate the average partition depth of the coding frame. Then, for different values of σ, the average partition depth of the CU at the edge was calculated, and the difference between the two was computed. A small difference indicates that too many non-edge pixels are detected as edge pixels, while a large difference suggests that the edge pixels are accurately extracted. For the coding frame *I(x, y*), the edge detection process is as follows. First, the image was smoothed using a Gaussian filter, and then, a Laplacian operator was applied to obtain the edge pixels *p(x, y)*, which are given by Equations (1) and (2). Here, *g(x, y)* is a 2D Gaussian function, and σ is the threshold value of the standard deviation of the Gaussian distribution, which was determined through experiments.

(1) $$p(x,y)=\nabla_{}^{2}[g(x,y)*I(x,y)]$$

The order of differentiation and convolution can be interchanged:

(2) $$\nabla_{}^{2}g(x,y)=\frac{1}{2\pi \sigma_{}^{4}}(\frac{x_{}^{2}+y_{}^{2}}{\sigma_{}^{2}}-2)\exp(-\frac{x_{}^{2}+y_{}^{2}}{2\sigma_{}^{2}})$$

**Figure 3:**
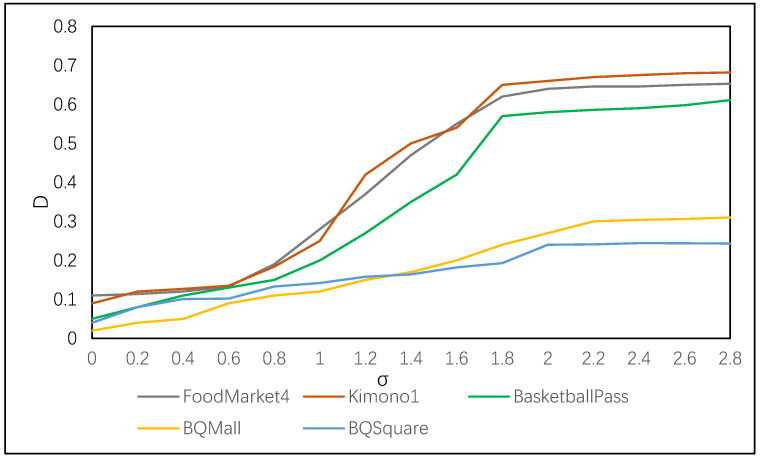
Depth difference ratios under different σ values.

We selected five video sequences with different resolutions and texture complexity from the JVET Common Test Conditions (CTC) [30], including FoodMarket4 (3840 × 2160), Kimono1 (1920 × 1080), BasketballPass (1920 × 1080), BQMall (832 × 480), and BQSquare (416 × 240). We calculated the difference between the average partition depth of the edge blocks detected at different values and the average partition depth of all coding blocks. The experimental results are shown in Figure 3. The horizontal axis represents the threshold value of σ, and the vertical axis represents the difference between the depth of coding unit (CU) partition at the edge and the depth of partition for all CUs within the frame at different sigma values.

**Figure 4:**
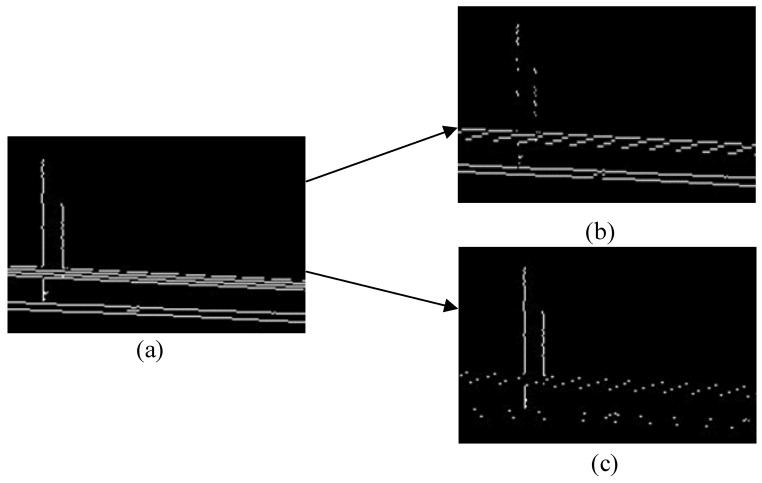
Image differentiation effect. (**a**) Original edge map. (**b**) Horizontal edges. (**c**) Vertical edges.

From Figure 4, we can observe that there are significant differences in the curves between different sequences, but the trend of change is consistent. When the value of σ is less than 0.6, the difference in partition depth between the edge coding blocks and the average depth is relatively small. This indicates that the edge image obtained by the LOG operator contains a large amount of noise and cannot accurately extract edges. When the value of σ is greater than 2.1, the difference in depth almost does not change with increasing σ, indicating that the extracted edges are relatively accurate. However, as the threshold value increases, some edges may be lost. Therefore, in this experiment, the threshold value for the standard deviation of the Gaussian distribution was set to 2.1, and the edges extracted under this threshold are shown in Figure 2c.

For CUs containing edges, we used edge feature values (*efv*s) to measure the directional characteristics of the edges. Compared to other methods, *efv*s are easier to calculate and more intuitively reflect the directional characteristics of the edges. The calculation process of *efv*s is as follows:

(3) $$efv=\frac{{efv}_{x}}{{efv}_{y}}$$

*efv_x_* and *efv_y_* are the edge feature values in the horizontal and vertical directions of the edge, respectively, and are calculated as follows.

We differentiated the edge image obtained by the LOG operator to make its features more obvious in the vertical and horizontal directions. The formula used is as follows.

(4) $$Dx(x,y)=\left|p(x,y-1)-p(x,y+1)\right|$$

(5) $$Dy(x,y)=\left|p(x-1,y)-p(x+1,y)\right|$$

where *Dx(x, y)* represents the horizontal edge pixels, *Dy(x, y)* represents the vertical edge pixels, *p(x, y)* represents the edge pixels obtained by the LOG operator, and *(x, y)* represents the pixel coordinates. The result is shown in Figure 4. As shown in Figure 4b, it is not difficult to see that although the optimized edge features in the horizontal direction are more obvious, there are still some pixels in the vertical direction. If we directly calculate the edge feature value in the horizontal direction at this time, there will be a large error. Similarly, if we calculate the edge feature value in the vertical direction at this time, it will also result in a large error. Considering the partition characteristics of VVC, we propose the following method to calculate the edge feature value.

Firstly, the current CU was divided into four sub-blocks along the horizontal direction, named *H_A_*, *H_B_*, *H_C_*, and *H_D_*, respectively, from top to bottom. Then, the number of horizontal edge pixels in each sub-block was calculated, as follows:

(6) $$H_{A}=\sum_{x=1}^{w}\sum_{y=1}^{\frac{h}{4}}\mathrm{Dx}(x,y)$$

(7) $$H_{B}=\sum_{x=1}^{w}\sum_{y=\frac{h}{4}}^{\frac{h}{2}}\mathrm{Dx}(x,y)$$

(8) $$H_{C}=\sum_{x=1}^{w}\sum_{y=\frac{h}{2}}^{\frac{3h}{4}}\mathrm{Dx}(x,y)$$

(9) $$H_{D}=\sum_{x=1}^{w}\sum_{y=\frac{3h}{4}}^{h}\mathrm{Dx}(x,y)$$

After obtaining the number of edge pixels for the four horizontal sub-blocks, we compared their sizes and recorded the largest number of pixels as *H_M_*. The second largest was recorded as *H_Sec_*. Similarly, we divided the CU into four vertical sub-blocks, from left to right, as *V_A_*, *V_B_*, *V_C_*, and *V_D_*, and then, calculated the number of vertical edge pixels in each sub-block. The calculation process is as follows:

(10) $$V_{A}=\sum_{x=1}^{\frac{w}{4}}\sum_{y=1}^{h}\mathrm{Dy}(x,y)$$

(11) $$V_{B}=\sum_{x=\frac{w}{4}}^{\frac{w}{2}}\sum_{y=1}^{h}\mathrm{Dy}(x,y)$$

(12) $$V_{C}=\sum_{x=\frac{w}{2}}^{\frac{3w}{4}}\sum_{y=1}^{h}\mathrm{Dy}(x,y)$$

(13) $$V_{D}=\sum_{x=\frac{3w}{4}}^{w}\sum_{y=1}^{h}\mathrm{Dy}(x,y)$$

Once we obtained the number of edge pixels in the four vertical sub-blocks, we took the largest pixel count as VM, and the second largest as V_Sec_. Then, we can calculated the edge feature value *efv_x_* in the horizontal direction and *efv_y_* in the vertical direction as follows.

(14) $${efv}_{x}=H_{M}+H_{\mathrm{Sec}}$$

(15) $${efv}_{y}=V_{M}+V_{\mathrm{Sec}}$$

We conducted experiments on the reference software VVC VTM-13.0 to determine the relationship between the final partition mode and *efv*. In the experiment, we continued to use the five video sequences in the Joint Video Exploration Team (JVET) Common Test Conditions (CTC), including FoodMarket4 (3840 × 2160), Kimono1 (1920 × 1080), BasketballPass (1920 × 1080), BQMall (832 × 480), and BQSquare (416 × 240). The first five frames of each sequence were encoded with the profiles of all the intra coding. We collected the statistical results of efv and the corresponding vertical and horizontal partition modes, which are shown in Table 1.

From Table 1, we can see that the percentages of vertical and horizontal partition modes vary with different sequences. We can also observe a clear relationship between partition modes and the value of *efv*. When *efv* is greater than 1, the number of horizontal edge pixels is greater than that of vertical edge pixels, and the edge features tend to be horizontal. Therefore, the CU partition tends to be horizontal, and the proportion of the horizontal partition increases with an increase in the ratio. When *efv* is less than 1, the number of vertical edge pixels is greater than that of horizontal edge pixels, and the edge features tend to be vertical. Therefore, the CU partition tends to be vertical, and the proportion of the vertical partition increases as *efv* decreases.

Based on the statistical data in Table 1, a fast CU decision-making scheme was designed based on the following rules. When the *efv* value is greater than the higher threshold Th, it means that the probability of horizontal direction is greater than that of vertical direction, and the current CU is more likely to have horizontal edges. In this case, we skip the vertical partition mode. Conversely, when the *efv* value is less than the lower threshold Tl, it indicates that the current CU is more likely to have vertical edges. Thus, we skip the horizontal partition mode. When *efv* is between Th and Tl, it indicates that the edge direction feature is not obvious. In this case, each CU partition has almost the same probability, and no partition mode is skipped in advance.

### 3.3. Early Termination of Simple-Texture Regions

**Figure 5:**
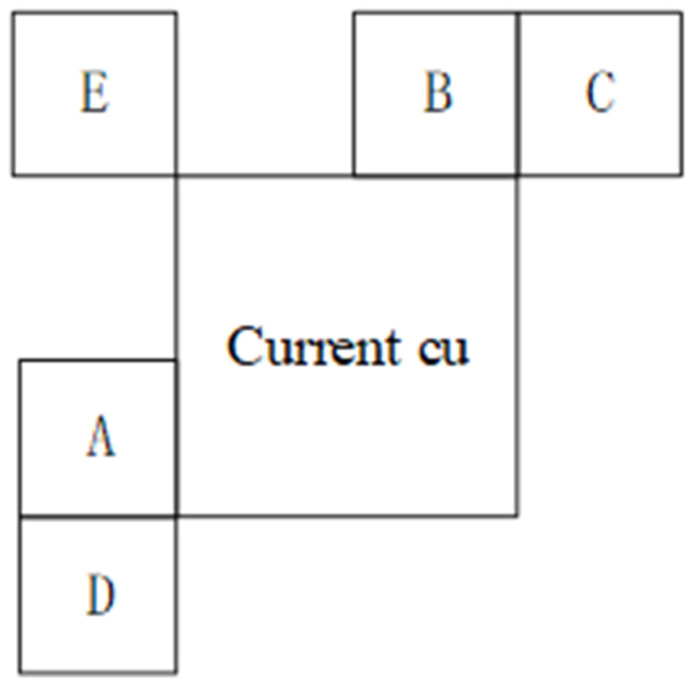
Adjacent coding unit (CU)s.

Simple-texture regions in CUs tend to favor larger partition sizes. We can terminate the partition process in advance. As shown in Figure 1, we find that the partition depth of CUs in simple-texture regions is very close to the partition depth of adjacent simple-texture region CUs, and the texture complexity of CUs decreases with an increase in partition depth. Therefore, we can use the partition depth and texture complexity of adjacent CUs as a basis for early termination of partition in simple-texture CUs. The distribution of adjacent CUs is shown in Figure 5.

We propose the following method to determine whether to terminate the partition process of a simple-texture-region CU in advance. First, we obtain the partition depth of the simple-texture-region CU. As shown in Figure 5, A–E are the adjacent CUs to the current CU. We refer to the partition information of A, B, and E to obtain the maximum division depth difference between adjacent CUs, and record the maximum division depth difference as *Dm*. The calculation formula of *Dm* is as follows:

(16) $$D_{m}=D_{max}-D_{min}$$

where *D_max_* and *D_min_* are the maximum and minimum partition depths of adjacent CUs, respectively. We record the current CU partition depth as CUdepth. If Dm is less than or equal to 1 and CUdepth is less than *D_max_*, then the CU partition continues. Otherwise, further judgment is made based on the texture complexity Ct to determine whether to terminate the partition. The calculation formula of Ct is as follows:

(17) $$C_{t}^{}=\frac{\sum_{i=x}^{x+w}\sum_{j=y}^{y+h}(p(i,j)-p_{average}^{}{)}^{2}}{w\times h}$$

where *p*(*i*, *j*) represents the pixel value at coordinates (*i*, *j*), and *P_average_* is the average pixel value of the CU. The calculation formula of *P_average_* is as follows:

(18) $$p_{average}^{}=\frac{1}{w\times h}\sum_{i=x}^{x+w}\sum_{j=y}^{y+h}p(i,j)$$

By calculating the texture complexity *Ct* of the current CU and its adjacent CUs, when the texture complexity (*Ctcu*) of the CU is less than or equal to the minimum texture complexity (*Ctmin*) of the adjacent CU, the segmentation process is terminated in advance.

### 3.4. Flowchart of the Proposed Algorithm

**Figure 6:**
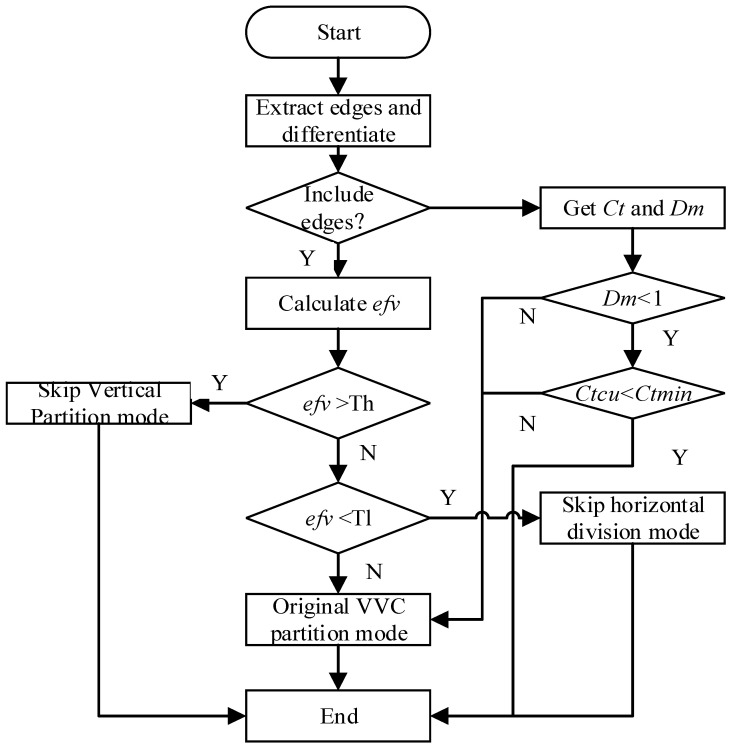
A flowchart of the proposed algorithm.

The flowchart of the algorithm process is shown in Figure 6. Firstly, the type of the current CU is determined using edge information. If it is a CU with a complex-texture region, the edge feature value *(efv*) of the current CU is calculated. We skip unnecessary partition modes for the current CU based on *efv*. If the *efv* value is greater than Th, the vertical partition mode is skipped. If the *efv* value is less than Tl, the horizontal partition mode is skipped. Otherwise, we consider that the directional features are not obvious, and do not skip any mode. For simple-texture regions that do not contain edges, we obtain the partition information of the current CU and its adjacent CUs. If the difference in partition depth between adjacent CUs does not exceed 1, we consider that the current CU and its adjacent CUs have a strong spatial correlation, and use spatial correlation to terminate partition. If the partition depth of the current CU is less than that of the adjacent CU, the partition process continues. Otherwise, we further compare the texture complexity of the current CU with that of the adjacent CU. If the texture complexity of the current CU is less than or equal to that of the adjacent CU, the partition process is terminated earlier.

## 4. Simulation Results

To evaluate the performance of our method, we conducted experiments on the reference software VVC VTM-13.0. In the experiment, 21 sequences were tested, which belong to six sequences with different resolutions recommended by JVET common test conditions. Four QPs of 22, 27, 32, and 37 were tested in the proposed algorithm. The experiments were conducted on a Windows 10 64-bit operating system, with an Intel(R) Core(TM) i5-10300H CPU @ 2.50 GHz. The performance of the algorithm was measured using *Ts* and BDBR, where Ts is calculated as follows:

(19) $$Ts=\frac{\mathrm{To}-Tp}{\mathrm{To}}\times 100\%$$

To represent the total encoding time of the original VVC standard, *Tp* represents the total encoding time of the proposed method, and BDBR represents the degree of loss in encoding performance. A smaller BDBR value indicates less compression performance loss.

Table 2 presents the experimental results of the proposed algorithm compared to the original VTM-13.0 platform under different parameters. When the experimental parameters Th and Tl are set to 0.6 and 1.5, respectively, the average coding time is reduced by 32.52%, with a BDBR loss of only 0.36%. For this case, the skip conditions for unnecessary partition modes are relatively strict with less time saving, and the impact on the coding performance can be ignored. When Th and Tl are set to 0.8 and 1.3, the average coding time is reduced by 54.08%, with a BDBR loss of 1.61%. Since more partition modes are skipped under this condition, more time saving is achieved with a slight increase in BDBR loss.

The method proposed in [15] saves 31.44% encoding time with a BDBR loss of 0.59%. Our method reduces the coding time by 32.52%, while maintaining better coding performance. In [15], the CU is divided into four sub-blocks, and the partition mode is skipped according to the complexity ratio of different combinations of the four sub-blocks. This method can achieve good results when the texture of the sequence is relatively simple. However, when the complex texture distribution of the CU is concentrated on one side, there is a possibility of misjudgment. Our method is more robust and can adapt to different situations of the encoded sequence. When the texture of the encoded sequence is simple, our method uses spatial correlation to terminate the partition process for CUs with a simple-texture area in advance. When the texture of the encoded sequence is complex, our method skips more non-optimal partition modes by utilizing edge features.

Compared with the methods proposed in [9,16], our proposed method achieves an additional time saving of 2.91% and 6.17% while maintaining better RD performance. The reason is that the methods in [9,16] require a large amount of data to train their models, and the performance of the method may be affected if the training data are insufficient or not comprehensive enough. The method we propose is based on an analysis of edge structure and spatial correlation, which requires lower computational complexity.

**Figure 7:**
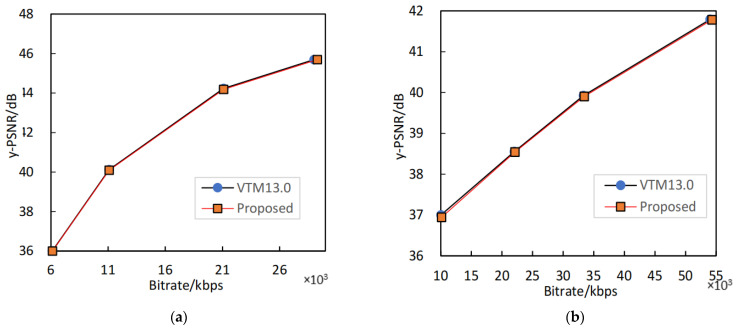
The comparison of RD performance between the proposed algorithm and the original encoder. (**a**) RD performance of BasketballDrill. (**b**) RD performance of BQTerrace.

To further illustrate the rate-distortion performance of our algorithm, we show the RD curves of the sequences in Figure 7. Figure 7a,b shows the RD curves for BasketballDrill and BQTerrace, respectively. The red curve represents the RD curve of the proposed method, and the black curve represents the RD curve of the original algorithm. In the worst case, the RD curve of the proposed method is slightly lower than that of the original encoder. In the best case, the RD curve of our proposed method almost coincides with that of the original encoder. Therefore, our algorithm effectively saves encoding time while maintaining high RD performance.

## 5. Conclusions

This paper proposes a low-complexity algorithm for VVC intra coding. The algorithm divides the encoding contents into two categories with edge features, complex-texture areas, and simple-texture areas. In the complex-texture areas, non-essential partition modes are skipped by analyzing the characteristics of the edge direction. In simple-texture areas, the partition process is terminated earlier based on spatial correlation. The algorithm is tested on the reference software VVC VTM-13.0 and achieves an average coding time saving of 54.08% with an increase of 1.61% BDBR.

## Figures and Tables

**Table 1:** Statistical analysis of partition modes under different edge feature values.

Encoding Sequence	Proportion of Vertical Division (%)	Proportion of Horizontal Division (%)
(a) FoodMarket4		
*efv* > 1	33	67
*efv* < 1	65	35
*efv* > 1.2	21	79
*efv* < 0.8	83	17
(b) Kimono1		
*efv* > 1	30	70
*efv* < 1	67	34
*efv* > 1.2	19	81
*efv* < 0.8	75	25
(c) BasketballPass		
*efv* > 1	21	79
*efv* < 1	72	28
*efv* > 1.2	19	81
*efv* < 0.8	75	25
(d) BQMall		
*efv* > 1	38	62
*efv* < 1	71	29
*efv* > 1.2	25	75
*efv* < 0.8	70	30
(e) BQSquare		
*efv* > 1	38	62
*efv* < 1	71	29
*efv* > 1.2	25	75
*efv* < 0.8	70	30

**Table 2:** Comparison of algorithm results.

Class	Sequence	Reference [9]		Reference [15]		Reference [16]		Algorithm in This Paper			
								Tl1 = 0.6, Th1 = 1.5		Tl1 = 0.8, Th1 = 1.3	
		BDBR (%)	Ts (%)	BDBR (%)	Ts (%)	BDBR (%)	Ts (%)	BDBR (%)	Ts (%)	BDBR (%)	Ts (%)
A1	Tango2	1.47	52.23	0.74	37.01	1.59	51.85	0.40	32.96	1.54	56.97
	Campfire	2.65	64.74	0.66	34.05	1.61	50.11	0.45	33.45	1.63	57.96
	CatRobatl	1.77	47.63	0.54	29.91	1.55	50.59	0.32	29.77	1.91	58.41
A2	DatLightRoat2	2.11	52.01	0.71	32.12	1.77	47.92	0.26	31.45	1.36	55.25
	ParkRunning3	1.32	50.12	0.68	32.11	1.99	54.33	0.27	29.45	1.51	49.07
	MarkPlace	1.91	55.21	0.55	34.15	1.86	48.11	0.36	33.11	1.77	55.11
B	Cactus	1.95	51.07	0.61	30.73	1.31	44.95	0.44	31.37	1.54	51.07
	BasketballDrive	2.25	62.01	0.74	34.48	1.42	48.33	0.32	35.21	1.56	49.72
	BQTerrace	2.07	54.07	0.62	30.85	1.49	46.16	0.27	34.32	1.32	50.01
C	RaceHorses	1.16	46.39	0.46	27.83	1.69	51.04	0.33	31.02	1.55	61.27
	BasketballDrill	2.01	46.19	0.40	26.55	1.52	51.18	0.40	34.21	2.17	66.21
	BQMall	2.15	53.23	0.65	33.79	1.44	46.95	0.48	30.38	1.54	56.02
	PartyScene	1.61	42.73	0.42	31.62	1.79	45.88	0.23	29.32	1.32	47.65
D	RaceHorses	1.33	43.75	0.55	30.17	1.24	48.33	0.24	31.41	1.49	50.21
	BasketballPass	2.33	43.85	0.70	30.53	1.18	45.17	0.28	31.87	1.37	45.95
	BQSquare	0.81	44.06	0.29	29.97	1.41	40.04	0.78	33.33	1.88	56.35
	BlowingBubbles	1.31	55.16	0.43	29.34	1.86	43.86	0.66	35.22	1.57	49.15
E	FourPeople	2.75	55.64	0.78	35.63	1.75	46.68	0.40	31.85	1.54	50.77
	Johnny	3.29	56.98	0.69	30.65	1.27	39.21	0.50	27.93	1.62	54.57
	KristenAndSara	2.51	57.19	0.59	31.38	1.63	49.82	0.36	32.66	1.62	45.91
	Average	1.94	51.17	0.59	31.44	1.56	47.91	0.36	32.52	1.61	54.08

## Data Availability

Data is unavailable due to privacy or ethical restrictions.
